# Effects of Nitrogen Accumulation, Transportation, and Grain Nutritional Quality and Advances in Fungal Endophyte Research in Quinoa (*Chenopodium quinoa* Willd.) Plants

**DOI:** 10.3390/jof10070504

**Published:** 2024-07-21

**Authors:** Linghong Li, Zhijun Jiang, Xinhui Yang, Yulai Zhang, Jianxun Huang, Jing Dai, Hafeez Noor, Xiangyun Wu, Aixia Ren, Zhiqiang Gao, Min Sun

**Affiliations:** 1 College of Agronomy, Shanxi Agricultural University, Taigu 030801, China; lilinghong00en@163.com (L.L.); jzj0430dd@163.com (Z.J.); 15638739408@163.com (X.Y.); wzdfl2022h@163.com (Y.Z.); 18203443065@163.com (J.D.); hafeeznoorbaloch@gmail.com (H.N.); rax_renaixia@163.com (A.R.); gaozhiqiang1964@126.com (Z.G.); 2 College of Agriculture Engineering, Shanxi Agricultural University, Taigu 030801, China; huangjianxun521@163.com; 3 Shanxi Jiaqi Agri-Tech Co., Ltd., Taiyuan 030006, China; quinoaking@icloud.com

**Keywords:** nitrogen fertilizer, quinoa, yield, grain quality, seed-endophytic bacteria

## Abstract

This study aims to understand the influence of nitrogen accumulation, fungal endophyte, yield, nitrogen use efficiency, and grain nutritional quality parameters on the yield of quinoa in some areas of China. The endophytic microbial community in plants plays a crucial role in plant growth, development, and health, especially in quinoa plants under different nitrogen fertilizer levels. The results from the present study indicated that appropriate nitrogen application significantly enhanced the nitrogen accumulation and yield of quinoa grains during maturity, increasing by 34.54–42.18% and 14.59–30.71%, respectively. Concurrently, protein content, amylose, total starch, ash, and fat content also increased, with respective growth rates of 1.15–18.18%, 30.74–42.53%, 6.40–12.40%, 1.94–21.94%, and 5.32–22.22%. Our constructed interaction network of bacterial and fungal communities revealed that bacteria outnumbered fungi significantly, and most of them exhibited synergistic interactions. The moderate increase in N150 was beneficial for increasing quinoa yield, achieving nitrogen use efficiency (NUE) of over 20%. The N210 was increased, and both the yield and NUE significantly decreased. This study provides novel insights into the impact of nitrogen fertilizer on quinoa growth and microbial communities, which are crucial for achieving agricultural sustainable development.

## 1. Introduction

Quinoa (*Chenopodium quinoa* Willd.), originating from the Andes of South America, is primarily distributed in Peru, Bolivia, Ecuador, and Chile; with a cultivation history spanning over 7000 years, it is revered by the Incas as the “mother of grains” [1]. Classified as a whole-nutrient functional food, quinoa is rich in various essential amino acids [2], proteins, vitamin E, and dietary fiber [3,4], boasting unique and extensive nutritional value [5]. Some research indicates that quinoa’s protein content surpasses corn (13.4%) and barley (11.0%), rivaling wheat (15.4%) [6]. Moreover, its balanced amino acid composition fulfills the needs of humans and infants. Additionally, quinoa is abundant in dietary fiber (7.0% to 9.7%), including both soluble and insoluble types, which greatly benefits human health. Internationally recognized for its nutritional value, the Food and Agriculture Organization of the United Nations [6] designates quinoa as the only single plant capable of fulfilling basic human nutritional requirements. International nutritionists refer to quinoa as a “super cereal” and recommend it as the perfect “whole-nutrient food” for humans [7].

Nitrogen, a crucial component of proteins, nucleic acids, chlorophyll, and certain hormones in plants, significantly impacts crop growth and yield [8]. However, nitrogen fertilizer application should be balanced; excessive application can lead to late maturity, lodging, and even yield reduction, while adequate application promotes growth [9,10]. Previous studies suggested that nitrogen fertilizer can regulate crop nutritional quality. Due to ecological variability, the nitrogen requirements of quinoa crops remain widely researched globally. For instance, moderate nitrogen fertilizer application not only promoted the growth of quinoa seedlings but also alleviated drought stress [11]. Previous studies have found that moderate nitrogen fertilizer application not only promoted the growth of quinoa seedlings but also alleviated drought stress [12]. Tiaoli No. 1 thrived best under a nitrogen application rate of 60 kg ha^−1^ with a base-to-topdressing ratio [13]. Nevertheless, domestic research on the effects of nitrogen application on nitrogen accumulation and translocation in quinoa plants, grain nutritional quality, and endophytic microbiota remains limited.

Endophytic bacteria refer to asymptomatic microorganisms that reside within plant tissues, forming symbiotic relationships with their hosts [14]. These microorganisms provide significant benefits to their host plants, including enhanced stress resistance, insect and disease resistance, productivity, and herbicide activity through the production of various substances [15]. This underscores the crucial role of endophytic bacteria in ecological communities, which are crucial for plant development, yield enhancement, and resilience.

With the rapid development of high-throughput sequencing technology, scientists have begun to explore the secrets of nitrogen fertilizer regulation on endophytic bacteria. They discovered that nitrogen fertilizer application rates and methods significantly influence the community structure and diversity of endophytic bacteria and fungi in plants [16]. Seeds treated with moderate nitrogen fertilization exhibited higher bacterial and fungal community diversity and abundance compared to excessive or no nitrogen application, with grain protein and amino acid contents increasing with nitrogen application. Under moderate nitrogen fertilization, the symbiotic network of seed endophytic microbial communities was more complex, enriching more potentially beneficial microbial strains, contrary to excessive nitrogen conditions. Additionally, nitrogen fertilizer regulation affects the interaction between endophytic bacteria and plants [17]. Under low nitrogen conditions, plants strengthen their symbiotic relationship with endophytic fungi, improving nitrogen absorption and utilization efficiency with the assistance of these fungi. This symbiotic relationship not only aids plants in surviving and reproducing under adversity but also provides new insights and methods for agricultural production. However, research on nitrogen fertilizer regulation of endophytic bacteria faces numerous challenges and unknowns, such as precise nitrogen application control, screening of endophytic bacterial strains with desirable traits, and evaluating their practical application in agriculture [18]. These issues require further exploration and research by scientists.

The objectives are to investigate the effects of N level for high-quality quinoa production in the region, reveal the mechanisms of nitrogen accumulation and translocation in quinoa plants and their relationship with quality, and provide technical support and theoretical foundations for the large-scale cultivation of quinoa, contributing to the provision of quality raw materials for the grain and food industries. Currently, quinoa research primarily focuses on yield, with limited reports on the effects of N on N accumulation and translocation in quinoa plants, grain quality, and especially endophytic microbiota.

## 2. Materials and Methods

### 2.1. Overview of the Test Site

The experiments were conducted in 2023 at the Taigu Experimental Base of Shanxi Agricultural University, Taigu District, Jinzhong City, Shanxi Province. The experimental site is located in the northeast of Jinzhong Basin in the eastern part of the Loess Plateau (37°42′ N, 112°53′ E). It is a continental semi-arid climate with four distinct seasons: cold and windy in winter, hot and less rainy in summer, and large temperature difference between day and night. The annual frost-free period is 175 days, the annual temperature is about 13 °C, the annual precipitation is about 458 mm, the annual average evaporation is 971 mm, and the annual sunshine time is 2500 h. The planting mode is one ripe a year or three cooked in two years. In 2023, the precipitation of quinoa during seeding and branching stage was 172.75 mm with an average temperature of 11.39 °C, the precipitation during branching and flowering stage was 91.05 mm with an average temperature of 17.79 °C, and the precipitation during flowering and maturing stage was 205.71 mm with an average temperature of 24.21 °C (Table S1). Regarding soil nutrients, on 15 April 2023, before sowing, the soil samples were collected by a five-point sampling method with a soil drill (diameter 40 mm), and the soil samples were fully mixed and sifted. The organic matter content, available nitrogen content, available phosphorus content, and available potassium content were determined. Organic matter was determined by potassium dichromate volume external heating method, alkali-hydrolyzed nitrogen was determined by alkali-hydrolysis diffusion method, soil available phosphorus was determined by ammonium fluoride–hydrochloric acid extraction–molybdenum antimony colorimetry, and soil available potassium was determined by ammonium acetate extraction–flame photometry. The basic nutrients of soil in 0–20 cm soil layer, the organic matter content was 15.19 g·kg^−1^, the alkali nitrogen content was 95.38 mg·kg^−1^, the quick phosphorus content was 34.66 mg·kg^−1^, and the quick potassium content was 210.66 mg·kg^−1^.

#### 2.1.1. Experimental Design

This experiment adopts a two-factor split-plot randomized block experimental design. The cultivar “JQ-307” provided by Shanxi Jiaqi Agricultural Science and Technology Co., Ltd, Taiyuan, China. Using the one-factor random design, the experiment included four nitrogen levels (N0, N90, N150, and N210) and sprinkler irrigation in the quinoa heading stage and flowering stage, with an irrigation volume 45 mm/time. The plot area was 2 × 7 = 14 m^2^, and the experiment was repeated 3 times. The sowing date was on 10 April 2023. The sowing method was manual, with a sowing depth of 1–2 cm and a row spacing of 40 cm. Nitrogen fertilizer in the form of urea was applied with a base–chase ratio of 5:5. Additionally, super-phosphate (16% P_2_O_5_) 100 k and 40 kg of potassium sulfate (50% K_2_O) were uniformly applied to the soil as a base fertilizer. When quinoa reached a 6–8 leaf period, the interval was 16 cm, and the number of fixed seedlings was 150,000 plants/hm^2^. During the growth period, artificial weeding and pest control were conducted, and the harvest was conducted on 22 August 2023. No herbicide was applied, all weeds were manually weeded, the quinoa was cut at six to eight leaves, and the grass was cut after that. The insecticide used was imidacloprid, purchased from Zhongnong Agricultural Facilities Co., Ltd, Beijing, China.

#### 2.1.2. Determination of Dry Mass

Dry mass: at each growth stage—seedling, branch, heading, flowering, filling, maturity—uniform and representative plants were taken from the field. During the seedling stage, the whole plant was taken. At the branch stage, the plant was divided into the main stem, branches, and leaves. During the heading, flowering, and filling stages, the plant was divided into four parts: main stem, branches, leaf, and ears. At the mature stage, the plant was divided into the main stem, branches, and ears. Each part was placed in a labeled kraft paper bag and processed by drying at 105 °C for 1 h, followed by drying at 80 °C to a constant weight after weighing.

Nitrogen content of plants: After crushing the dry samples of each organ obtained during each growth period, the nitrogen content was determined by the H_2_SO_4_-H_2_O_2_–indophenol blue color method.

#### 2.1.3. Yield and Components

For yield and components, when 80% of quinoa leaves were yellow, and some began to fall off, 20 evenly grown plants were taken from each cell, threshed, dried, and converted into the actual yield. To determine spike length and width, spike length was directly measured with a tape measure (in cm), and spike width was measured with a vernier caliper (in mm) to harvest all cell grains. For the determination of 1000 grain weight, 1000 grains were counted and weighted, with 3 replicates for each treatment.

Starch was determined using the double-wave colorimetric method [19].

Protein was determined using the national standard of the People’s Republic of China, (GB5009.5—2016), which outlines the determination of fats in foods using national food safety standards [20].

Regarding amino acid, quinoa grains were sent to Panomic Biomedical Technology Co., Ltd, Suzhou, China, for determination using a ZORBAX Eclipse XDB–C18 column (4.6 × 150 mm, Agilent, Santa Clara, California, USA).

Fat was determined by the National Standard of the People’s Republic of China (GB 5009.6-2016) using the Cereal Fat Soxhlet Extractor Method [21].

Ash was determined according to the national standard of the People’s Republic of China (GB5009.4—2016), which specifies the National Food Safety Standard for the determination of ash in food [22].

The nitrogen-related indicators were calculated as follows:

Plant nitrogen accumulation (kg·ha^−1^) = plant dry matter weight×nitrogen content;

Nitrogen accumulation after flowering (kg·ha^−1^) = nitrogen accumulation at maturity stage—nitrogen accumulation at flowering stage;

Contribution rate of nitrogen accumulation to grain after flowering (%) = nitrogen accumulation after flowering/nitrogen accumulation in grain × 100%;

Nitrogen uptake efficiency (kg·kg^−1^) = nitrogen accumulation/nitrogen application;

Nitrogen use efficiency (kg·kg^−1^) = grain yield/nitrogen accumulation of plants.

Excel 2010 (Microsoft Inc., Redmond, WA, USA, 2010) was used for data entry and collation, DPS 9.01 was used for ANOVA, and the OriginPro 2021 (64-bit) version 9.8.0.200 software was used for graphing.

#### 2.1.4. Methods for Determination of Endophytic Bacteria in Grains

Mature seeds were harvested from each plot, sealed in sterile nylon bags, and stored at 4 °C until further study. In order to exclude the influence of microflora on the surface of seeds, 3 replicates (~0.5 g seeds) of seeds treated with different levels of nitrogen were randomly selected, and the seeds were disinfected on the surface before sending for testing. The specific disinfection process is as follows: The seeds were washed twice with sterile water, then rinsed with 70% (*v*/*v*) ethanol for 30 s; the ethanol was discarded, treated with 5% sodium hypochlorite again for 2 min, and rinsed with 70% (*v*/*v*) ethanol for 30 s. Finally, it was washed 7 times with sterile water and dried in a sterile environment before grinding.

The grain sample was taken out of the refrigerator, and the appropriate amount of sample (between 0.2 and 0.5 g) was taken for grinding as soon as possible. The Mag Beads Fast DNA Kit for Soil (116564384) (MP Biomedicals, CA, USA) kit was used to extract nucleic acid. The highly variable V5V7 region of the bacterial 16S rRNA gene and the fungal ITS1 region were selected for sequencing. Primers are shown in Table S2. PCR conditions were as follows: 98 °C detection for 3 min, 27 cycles (98 °C detection for 30 s, 53 °C detection for 30, 72 °C detection for 45 s), and 72 °C detection for 5 min. Quantification of PCR products on a Microplate reader (BioTek, FLx800, Winooski, Vermont, USA) was performed using the Quant-iT PicoGreen dsDNA Assay Kit, followed by mixing samples according to the amount of data required for each sample. TruSeq Nano DNA LT Library Prep Kit (Illumina, Foster City, CA, USA) was used to construct the library. A 1 μL library was taken, and a 2100 quality inspection was conducted on the library using an High Sensitivity DNA Kit (Agilent, USA) on the Agilent Bioanalyzer machine. Quant-iT Pico Green dsDNA Assay Kit (Thermo Fisher Scientific, USA) was used to quantify the library on QuantiFluor (Promega, Madison, Wisconsin, USA). For qualified libraries, we performed 2 × 250 nbp double-ended sequencing using the NovaSeq 6000 SP Reagent Kit (500 cycles, Illumina, USA) on the Illumina NovaSeq machine (Illumina, USA).

#### 2.1.5. Data Quality Control and Analysis

Microbiome biological information was analyzed using QIIME2 version 2019.4 [23]. By using the Greengenes database, the characteristic sequences of ASV were compared with the reference sequences in the database to obtain the taxonomic information corresponding to each ASV. At the same time, R software (4.4.0) was used to draw the identification results of each sample at each classification level into a bar chart to visually compare the differences in ASV number and classification status identification results of different samples [24].

The Alpha diversity index was analyzed using QIIME2 software (2019.4), the Chao1 index and Shannon index were calculated for each sample, and box plots were drawn to compare the richness and evenness of ASV among different samples. Using R software (4.4.0) and QIIME2 software (2019.4), Beta diversity analysis was performed using UniFrac distance metrics to investigate changes in microbial community structure between samples, visualized by principal coordinate analysis (PCoA) [25].

According to the results of ASV classification and taxonomic status identification, the specific species composition of each sample at each taxonomic level can be obtained. The number of microbial groups contained in different samples at different classification levels was compared, and then R software (4.4.0) was used to draw the above data into a bar chart to visually compare the number of taxons of different samples at the same level. QIIME2 software (2019.4) was used to obtain the composition and abundance tables of each sample at the six taxonomic levels of phyla, class, order, family, genus, and species, and the analysis results were presented by histogram.

PERMANOVA (Adonis/PERMANOVA analysis) was used to evaluate the significance of differences in microbial community structure between groups. Linear discriminant analysis effect size (LEfSe) method was used to detect taxa with rich differences between groups [26].

The construction of the association network adopted the SparCC analysis method, and the pseudocount value in SparCC was set to 10^−6^. The R value of the correlation coefficient was determined by the RMThreshold packet based on random matrix theory, and the *p*-value of significance was calculated [27]. On the basis of correlation coefficient and significance, we built a modular network in which nodes represent ASVs, and the lines between nodes represent the correlation between ASVs. R language and ggraph data packages were used to visualize the correlation network. For analysis of the correlation network, Mothur software 1.48.1 was used to calculate the Spearman correlation coefficient between bacterial and fungal abundance, and the correlation network was constructed for the correlation information rho > 0.8 and *p* value < 0.01 [28].

## 3. Results

### 3.1. Nitrogen Accumulation and Translocation in Quinoa Plants

With the increase in nitrogen application, the amount of nitrogen trans located before flowering in quinoa initially increases and then decreases, reaching a peak nitrogen application rate of N150. Post-flowering nitrogen accumulation and its contribution to the grains show a gradual increase, peaking at N210. The grain nitrogen accumulation also follows a trend of first rising and then falling, with the highest value observed at N150 (Table 1). Compared to the no-nitrogen application, nitrogen application significantly increases grain nitrogen accumulation by 34.54–42.18%, particularly at N150, which shows a significant difference from N90 but not from N210. This indicates that nitrogen application enhances nitrogen absorption and translocation in quinoa plants. N150 promotes nitrogen translocation before flowering, while increasing the rate to N210 further promotes post-flowering nitrogen accumulation, albeit with less pronounced effects.

#### 3.1.1. Effects of Nitrogen Application Rates on Quinoa Yield and Nitrogen Use Efficiency

**Figure 1:**
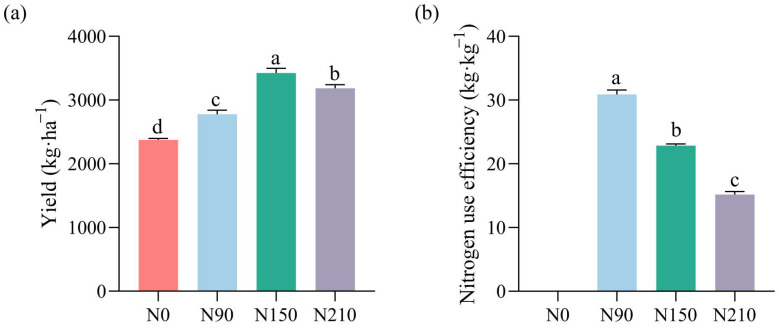
Effects of nitrogen application rates on quinoa yield and nitrogen use efficiency. Different small letters mean significant difference at *p* < 0.05 using the Tukey multiple-comparison test (**a**,**b**).

With the increase in nitrogen application, the yield of quinoa initially rises and then falls, reaching a peak at the level of N150, while the nitrogen use efficiency (NUE) gradually decreases, as shown in (Figure 1). The yield of quinoa ranges from 2373.83 to 3425.95 kg·ha^−1^. The results compared to N0 show that nitrogen application significantly increases the yield by 14.59–30.71%, especially at the rate of N150. The NUE of quinoa ranges from 15.17 to 30.88 kg·kg^−1^, particularly at the rate of N90. This indicates that a moderate increase in nitrogen application to N150 was beneficial for increasing quinoa yield, achieving an NUE of over 20%. However, when the rate was increased to N210, both the yield and NUE significantly decreased.

#### 3.1.2. Effects of Nitrogen Application Rates on Nutritional Quality of Quinoa Grains

The protein, amylose, amylopectin, total starch, ash, and fat contents in quinoa grains at maturity initially rise and then fall, peaking at N150 (Table 2). Compared to no nitrogen application, nitrogen application increases the protein content by 1.15–18.18%, amylose content by 30.74–42.53%, total starch content by 6.40–12.40%, ash content by 1.94–21.94%, and fat content by 5.32–22.22%, especially at a rate of N150. Significant differences were observed in protein, ash, and fat contents in the N150 treatment compared to the N0 and N90 treatments. Amylose and total starch contents at N150 also showed significant differences from other nitrogen rates, while the differences in amylopectin content among all treatments were not significant. This indicates that nitrogen application, particularly at N150, improves the nutritional quality of quinoa grains.

When the nitrogen application rate increases, the total amino acid and non-essential amino acid contents in quinoa grains at maturity increase, peaking at N210, while essential amino acids initially rise and then fall, peaking at N150 (Table 3). Compared to no nitrogen application, nitrogen application increases the contents of essential amino acids, non-essential amino acids, and total amino acids by 1.81–11.54% and 3.09–14.04%, respectively. The essential amino acids valine, methionine, histidine, and tryptophan particularly increase at N150, showing significant differences from other treatments. Non-essential amino acids and total amino acids particularly increase at N210, showing significant differences from the N0 and N90 treatments but not from the N150 treatment.

#### 3.1.3. Effects of Nitrogen Application on Endophytic Microflora of Quinoa Grain

**Figure 2:**
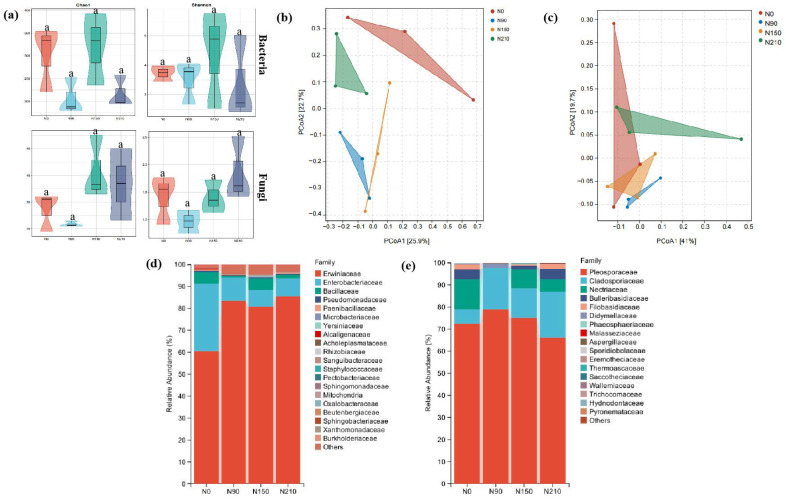
Alpha and beta-diversity of seed endophytic microbial communities in rice under different N application rates. Note: (**a**) Alpha diversity for bacterial and fungal communities. Treatments with the same letters are not significantly different (*p* < 0.05; Dunn’s multiple-comparison test). Beta-diversity principal coordinate analysis based on unweighted UniFrac distances, showing the seed endophytic bacterial (**b**) and fungal (**c**) community structures under different N application rate treatments. Seed endophyte microbial (bacterial (**d**) and fungal (**e**) community compositions under different N application rates at the family level.

To delve deeper into the impact of various nitrogen fertilizer treatments on the endophytic microbial communities within quinoa seeds, we conducted DNA extraction from surface-sterilized quinoa seeds and employed the HiSeq high-throughput sequencing technology to determine their microbial community structures. After rigorous quality filtering and chimera removal, we successfully obtained a total of 1,428,589 bacterial sequences (averaging 119,049 per sample) and 1,429,867 fungal sequences (averaging 119,155 per sample) from 12 quinoa seed samples. After further elimination of chloroplast and mitochondrial sequences, we precisely identified 1565 bacterial ZOTUs and 189 fungal ZOTUs, with a sequence similarity of up to 100%. As nitrogen application increased, we observed a trend of initial increase and subsequent decrease in the alpha diversity (measured by Chao1 and Shannon indices) of both bacterial and fungal communities, peaking specifically under the N150 treatment (Figure 2a). Upon conducting beta diversity analysis using the PCoA method based on Unifrac distances, we found no significant differences in the bacterial and fungal community structures among different nitrogen fertilizer treatments (Figure 2b,c). Within the bacterial community, Proteobacteria (88.6–99.7%) and Firmicutes (2.04%, 5.25%) dominated. At the family level, the relative abundance of Erwiniaceae was most prominent, especially after nitrogen fertilization (>80%), showing a significant increase compared to the non-fertilized treatment (60.52%), and reaching its highest abundance under the N210 treatment (85.57%). On the other hand, Bacillaceae exhibited the highest abundance when no nitrogen was applied (5.31%) but significantly decreased under N90 and N210 treatments while remaining largely unchanged under the N150 treatment (5.15%) (Figure 2d). Among the fungal community, Pleosporaceae was the most abundant, accounting for 65.92% to 78.92% and showing an initial increase and subsequent decrease with increasing nitrogen application. Cladosporiaceae had the lowest abundance under non-fertilized conditions (6.60%), but its abundance increased significantly after nitrogen fertilization, peaking under the N90 condition (Figure 2e).

**Figure 3:**
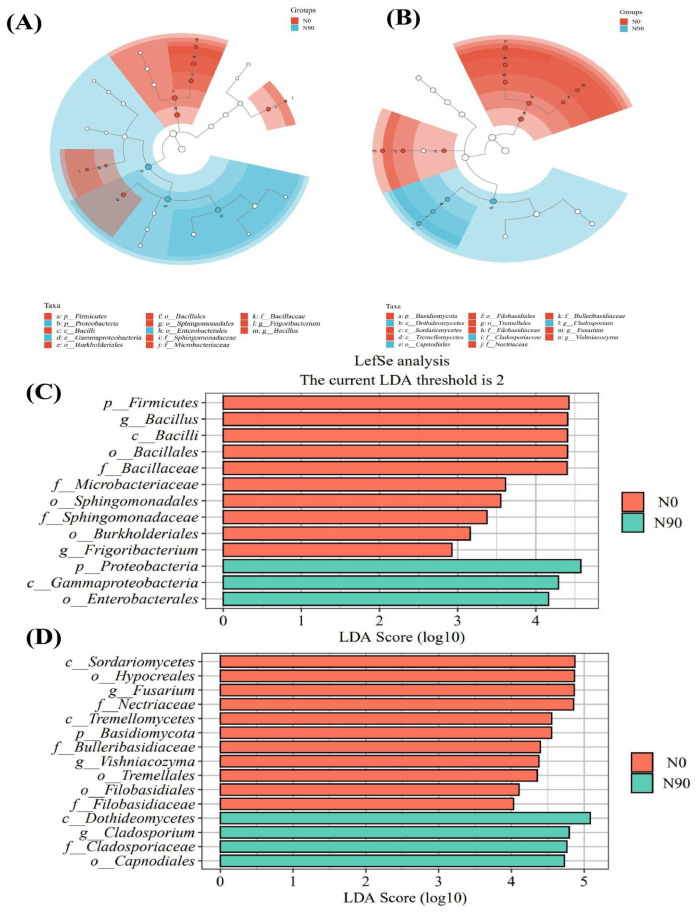
Linear discriminant analysis effect size (LEfSe) of bacterial (**A**) and fungal (**B**) taxa with an LDA score (log 10) > 2.0 in quinoa endophyte communities under different N application rates. Cladograms represent the phylogenetic distribution of microbial lineages. Circle paths indicate phylogenetic levels from kingdom to genus; each node represents one taxon. Visualization of differential features among treatments for bacterial (**C**) and fungal (**D**) microbial communities ranked by effect size.

To identify statistically significant microbiota characteristics among different groups, we employed LEf Se analysis (Linear Discriminant Analysis Effect Size), focusing specifically on treatments with LDA scores exceeding 2.0, and conducted an in-depth comparison of the differences in endophytic microbiota between the N0 and N90 groups. The results revealed significant differences in bacterial communities between nitrogen application and no nitrogen application treatments. Specifically, under the N0 level, multiple microbiotas, such as the Bacilli class, Bacillales order, Bacillaceae family, and Bacillus genus under the Firmicutes phylum (LDA > 4.40); Frigoribacterium genus under the Microbacteriaceae family (LDA > 2.93); Sphingomonadaceae family under the Sphingomonadales order (LDA > 3.37); and Burkholderiales order (LDA > 3.16), showed significant enrichment in relative abundance. Under the N90 level, the relative abundance of the Gammaproteobacteria class and Enterobacterales order under the Proteobacteria phylum was significantly enriched (LDA > 4.16) (Figure 3A,C). For fungal communities under the N0 level, the Tremellomycetes class, Tremellales order, Bulleribasidiaceae family, and Vishniacozyma genus under the Basidiomycota phylum (LDA > 4.36), as well as the Filobasidiaceae family under the Filobasidiales order (LDA > 4.03), displayed active fungal activities. While under the N90 level, the Capnodiales order, Cladosporiaceae family, and Cladosporium genus under the Dothideomycetes phylum stood out particularly (LDA > 4.73) (Figure 3B,D). Further exploration of the functions of these bacterial microbiotas revealed that the Proteo bacteria class, Gammaproteobacteria order, and Enterobacterales order under the Bacteria phylum, as important members of the quinoa endophytic microbiota, may play vital roles in promoting plant growth, enhancing stress resistance, participating in nutrient cycling, and improving soil structure. The Ascomycota phylum, Dothideomycetes class, Capnodiales order, Cladosporiaceae family, and Cladosporium genus among fungi also exhibit multifaceted functions in the endophytic microbiota of seeds. They contribute to plant growth and soil quality improvement, participate in bioremediation, and enhance plant disease resistance.

**Figure 4:**
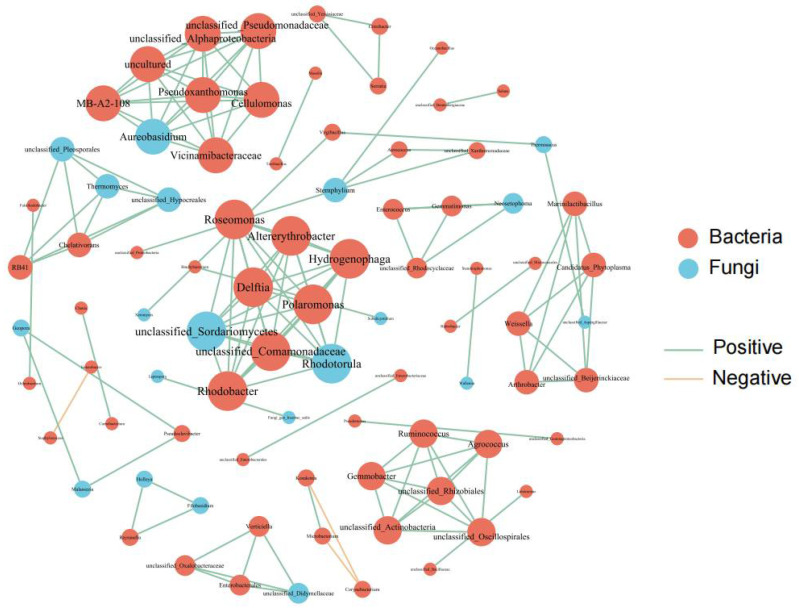
Co-occurrence networks of bacterial and fungal communities in seed endophyte communities. Node size is proportional to relative abundance.

To deeply explore the co-occurrence patterns of endophytic microbial communities in quinoa seeds, we conducted a network analysis of bacterial and fungal communities. The main nodes of the bacterial network came from Pantoea, Bacillus, and Pseudomonas. From the graph, we can clearly see that the proportion of bacteria shows a trend of first increasing and then decreasing as the nitrogen application rate increases, and the bacterial flora under N150 treatment appears particularly rich. The fungal network is mainly composed of Alternaria, Vishniacozyma, and Thermoascus (Figure S1). To explore the interrelationships between seed endophytic microbial communities, an interaction network between the two was constructed without grouping (Figure 4). The number of bacteria in the seed endophytic microbial community was significantly dominant, and most of the relationships between bacteria and fungi were in harmonious synergistic states, with only a few bacteria showing subtle antagonistic relationships. Among the bacterial communities, bacterial families such as Roseomonas, Altererythrobacter, and Rhodobacter occupy central positions due to their high abundance, playing a pivotal role in the interaction network. In the fungal community, fungi such as Rhodotorula, Sordariomycetes, and Aureobasidium are also prominent due to their high abundance, playing key roles in the interaction network.

### 3.2. Correlation Analysis of Nitrogen Accumulation and Movement, Yield, Grain Quality, and Endophytic Flora

**Figure 5:**
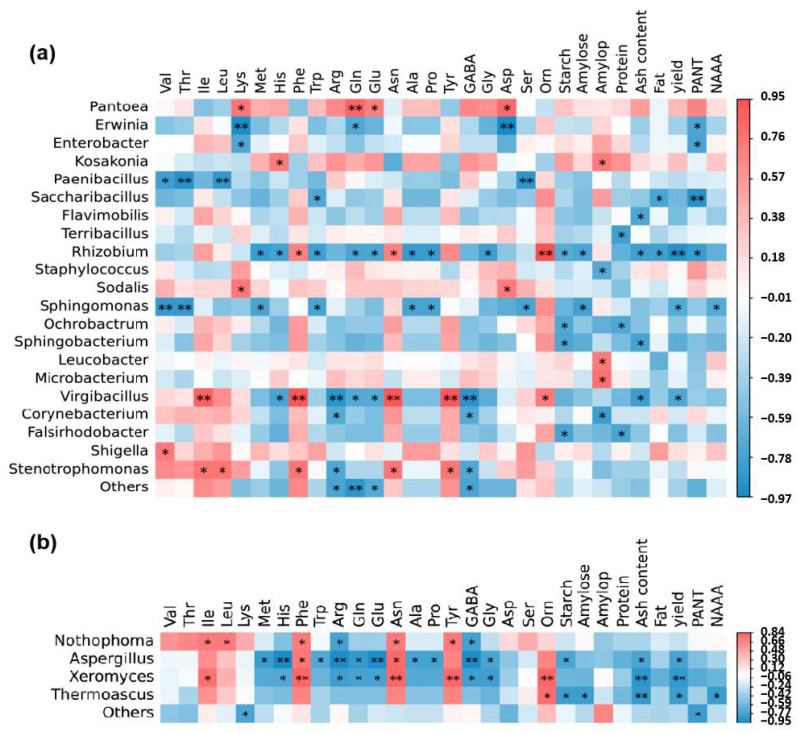
Correlation relationship between grain trait and endophytic bacterial (**a**) and fungal (**b**) taxa. The Spearman’s rank correlation coefficients and their corresponding *p*-values were calculated based on comparisons of the top 30 relative abundant genus and grain quality traits. * indicates significance at *p* < 0.05, ** indicates significance at *p* < 0.01. Asp (aspartic acid), Thr (threonine), Ser (serine), Glu (glutamic acid), Gly (glycine), Ala (alanine), Cys (cysteine), Val (valine), Met (methionine), Ile (isoleucine), Leu (leucine), Tyr (tyrosine), Phe (phenylalanine), Lys (lysine), His (histidine), Arg (arginine), Pro (proline), Starch (the total starch content), Amylose (the amylose content), Amylop (the amylopectin content), Protein (the protein content), Ash content (the ash content), Fat (the fat content), yield, PANT (N translocation before AS), NAAA (N accumulation after AS).

To elucidate the intricate connections between (NUE) crop yield, grain quality, and the endophytic microbial communities, we crafted correlation matrices utilizing Spearman coefficients, focusing on the prominent microbial genera (Figure 5). Within the bacterial realm, PANT displayed a negative correlation with the relative abundance of Erwinia, Enterobacter, Saccharibacillus, and Rhizobium. Yield, on the other hand, exhibited a marked negative association with Rhizobium, Sphingomonas, and Virgibacillus. Multiple amino acids in quinoa seeds exhibited both positive and negative correlations with various bacterial genera. For instance, valine and threonine were inversely related to Paenibacillus and Sphingomonas, whereas lysine, glutamine, glutamic acid, and aspartic acid positively correlated with Pantoea. Total starch content was negatively correlated with Rhizobium, Ochrobactrum, Sphingobacterium, and Falsirhodobacter. Amylopectin content showed a positive correlation with Kosakonia, Leucobacter, and Microbacterium but a negative one with Staphylococcus and Corynebacterium. Grain protein content, meanwhile, negatively correlated with Terribacillus, Ochrobactrum, and Falsirhodobacter. Ash and fat contents were negatively associated with diverse bacterial genera, with both exhibiting a significant negative correlation with Rhizobium (Figure 5a). For the fungal community, isoleucine, leucine, phenylalanine, asparagine, and tyrosine displayed positive correlations with Nothophoma, whereas arginine and GABA exhibited the opposite trend. Histidine, arginine, glutamine, glutamic acid, GABA, and glycine were negatively correlated with Aspergillus and Xeromyces. Total starch content was negatively correlated with Aspergillus and Thermoascus. Both ash content and yield negatively correlated with Aspergillus, Xeromyces, and Thermoascus. Additionally, NAAA displayed a negative correlation with Thermoascus. These intricate relationships highlight the vital role of endophytic communities in shaping crop productivity and grain quality (Figure 5b).

## 4. Discussion

### 4.1. Effects of Nitrogen Fertilizer on Nitrogen Accumulation, Transport Crop Quality

Appropriate application of nitrogen fertilizer plays an important role in promoting crop growth and improving NUE and yield. In this study, N150 was beneficial to the increase in quinoa yield, and the NUE was more than 20%, but the yield and NUE decreased significantly when the nitrogen application rate was increased to N210. It was confirmed that proper nitrogen application had positive regulating effects on plant growth and yield, while excessive nitrogen application had a negative effect [29]. Endophytic flora within plant tissues plays a pivotal role in nitrogen accumulation, translocation, and, ultimately, yield enhancement. Our research indicates a negative correlation between nitrogen translocation and the relative abundances of Erwinia, Enterobacter, Saccharibacillus, and Rhizobium within the bacterial communities. Notably, the yield of quinoa exhibited a significant negative correlation with Rhizobia, Sphingomonas, and Virgibacillus, highlighting the influence of endophytic bacteria on nitrogen translocation and yield augmentation in quinoa. Previous studies have revealed that endophytic bacteria can augment plants’ nitrogen absorption capacity. This augmentation might occur through mechanisms such as enhanced root growth, improved soil structure, or direct mediation of nitrogen conversion processes. Specifically, endophytic bacteria like Azotobacter and Azospirillum have been found to fix atmospheric nitrogen in the rhizosphere or plant tissues, converting it into a form readily absorbable by plants. Other studies have demonstrated that plants inoculated with these endophytic bacteria exhibit accelerated growth, greater biomass accumulation, and higher yields, partly attributed to the promotion of nitrogen uptake and utilization in plants by these endophytes [30].

### 4.2. Interactions of Endophytic Bacteria in Seeds and Their Effects on Plant Growth and Nutritional Quality

The endogenous microflora of seeds have a profound influence on plant growth and nutritional quality. Bacteria and fungi provide plants with rich nutrients such as nitrogen, phosphorus, and potassium by breaking down organic matter. These elements are essential for plant growth, promote root development, enhance stress resistance, and promote nutrient uptake. In addition, bacteria and fungi can produce growth hormones and other beneficial substances that further promote the growth and development of plants [31,32]. The adaptability of bacterial mutants through genome-wide analysis was studied, along with the interactions between bacteria and fungi. This research could give us a deeper understanding of how bacteria and fungi interact in the endogenous microbiota of seeds [33]. Previous studies have highlighted the importance of using microbiomes to study bacteria–fungus interactions and explored the potential applications of these interactions in promoting plant growth. These studies may contain information on how these microorganisms influence plant growth and nutrient quality in the seed’s inner sphere. The results of this study showed that the number of bacteria in seed endophytic flora was significantly higher than that of fungi, and most of the bacteria and fungi showed synergistic effects. In addition, there was a significant correlation between nutritional quality and the relative abundance of dominant bacteria and fungi genera. Streptomyces and acidomyces amycolatopsis are endophytic species with a high abundance of roots. The genus Streptomyces and the genus armatoides amycolatopsis are widely distributed in the soil and are important producers of resistant vegetative bacteria. A chain was isolated from leek by Igarashi [34]. Fungi, which can produce alkaloid active compounds such as 6-prenylindole, are effective in controlling the growth of fusarium oxysoorum after fermentation [35]. In 1956, amycolatopsis species were isolated from Indonesian soil during a soil screening project. Vancomycin, an important clinical glycopeptide antibiotic, was isolated from the fermentation broth of amycolatopsis orientalis [36,37].

### 4.3. Nitrogen Fertilizer Management Strategies to Ensure High Yield and Quality

In the pursuit of high yield and quality of quinoa, promoting the healthy development of endogenous beneficial microbial communities is a crucial strategy for achieving agricultural sustainability. Nitrogen fertilizer, as an indispensable nutrient source in agricultural production, plays a pivotal role in optimizing its management strategy to achieve this goal [38]. Firstly, a thorough understanding of the nitrogen fertilizer demand patterns during the growth process of quinoa is essential. As a crop, quinoa’s nitrogen requirement varies throughout its growth stages. Therefore, it is necessary to accurately assess the nitrogen fertilizer needs of quinoa through scientific methods such as soil testing and plant analysis and formulate precise nitrogen fertilization plans accordingly. Secondly, optimizing nitrogen fertilizer management strategies requires attention to the application methods and timing of nitrogen fertilizer. Traditional nitrogen fertilizer application methods often lead to fertilizer loss and environmental pollution [39,40]. Thus, we can consider adopting new nitrogen fertilizer products such as slow-release and controlled-release nitrogen fertilizers, as well as water-saving irrigation techniques like drip irrigation and sprinkler irrigation, to reduce nitrogen fertilizer loss and waste and improve nitrogen utilization efficiency. Simultaneously, we need to reasonably arrange the timing of nitrogen fertilizer application based on the growth cycle of quinoa and climatic conditions, ensuring that nitrogen fertilizer is fully utilized when quinoa needs it most [41]. Moreover, promoting the healthy development of endogenous beneficial microbial communities is also an important aspect of achieving agricultural sustainability. Endogenous beneficial microorganisms can assist quinoa in nutrient absorption and utilization, enhancing its disease resistance and stress tolerance. Therefore, we can increase the number and activity of endogenous beneficial microorganisms in the soil by applying organic fertilizers and using biological bacterial fertilizers. Additionally, we can create favorable conditions for the growth of endogenous beneficial microorganisms through agricultural management measures such as reasonable crop rotation and intercropping [42]. Optimizing nitrogen fertilizer management strategies to ensure the high yield and quality of quinoa while promoting the healthy development of endogenous beneficial microbial communities is an effective approach to achieving agricultural sustainability. This requires continuous exploration and innovation in practice, seeking more scientific and reasonable nitrogen fertilizer management strategies to promote the sustainable development of agriculture. Authors should discuss the results and how they can be interpreted from the perspective of previous studies and the working hypotheses. The findings and their implications should be discussed in the broadest context, and possible future research directions may also be highlighted.

## 5. Conclusions

Different N application treatments not only regulate nitrogen accumulation, transport efficiency, yield, and nitrogen use efficiency of quinoa plants but also affect the changes in grain nutrition quality and endophytic flora of quinoa plants. The nutritional quality of quinoa could be improved with a moderate nitrogen ratio. The diversity and abundance of bacteria and fungi in quinoa seeds treated with moderate nitrogen fertilization were higher than those treated with excessive or no nitrogen fertilization. The interaction network of bacterial and fungal communities was constructed, and the number of bacteria was significantly more than that of fungi, and most of them showed synergistic effects. The nitrogen accumulation, yield, and nutritional quality of quinoa were significantly correlated with the relative abundance of dominant genera of bacteria and fungi. This study provided an effective way for us to optimize N management strategies, ensure high yield and quality of quinoa, promote the healthy development of endogenous beneficial microbial communities, and realize sustainable agricultural development. Currently, quinoa research primarily focuses on yield, with limited reports on the effects of N on N accumulation and translocation in quinoa plants, grain quality, and especially endophytic microbiota.

## Figures and Tables

**Table 1:** Effects of nitrogen fertilization on post-flowering nitrogen accumulation and translocation in quinoa.

Nitrogen Application	Grain Nitrogen Accumulation (kg ha^−1^)	Pre-Flowering Nitrogen Translocation (kg)	Pre-Flowering Contribution to Grain (%)	Post-Flowering Nitrogen Accumulatio (kg)	Post-Flowering Contribution to Grain (%)
N0	63.23 c	41.86 c	66.18 a	21.38 c	33.82 c
N90	96.60 b	63.23 b	65.24 a	33.37 b	34.76 c
N150	109.36 a	69.67 a	63.65 b	39.69 a	36.35 b
N210	106.19 a	64.51 b	60.87 c	41.67 a	39.13 a

Note: Different small letters in the same column mean significant difference at *p* < 0.05 using the Tukey multiple-comparison test.

**Table 2:** Effect of nitrogen application rates on the nutritional quality of quinoa grains.

Nitrogen Application	Protein (g/100 g)	Amylose (%)	Amylopectin (%)	Total Starch (%)	Ash (g/100 g)	Fat (g/100 g)
N0	12.32 b	0.10 c	0.47 a	57.38 c	3.97 b	4.33 b
N90	12.47 b	0.14 b	0.47 a	61.30 b	4.04 b	4.58 b
N150	15.06 a	0.17 a	0.48 a	65.49 a	5.08 a	5.57 a
N210	13.34 ab	0.14 b	0.47 a	61.59 b	5.02 a	4.68 b

Note: Different small letters in the same column mean significant difference at *p* < 0.05 using the Tukey multiple-comparison test.

**Table 3:** Effect of nitrogen application rates on the amino acid composition of quinoa grains at maturity.

**Nitrogen Application**	**Essential Amino Acids (μg/g** **)**													
	**TAA**	Val *	Thr *	Ile *	Leu *	Lys *	Met *	His *	Phe *	Trp *	**EAA**	**EAA** **/TAA**	--	
N0	3669.31 b	68.10 c	28.69 d	51.27 b	62.74 b	130.51 b	34.64 b	193.73 c	55.22 b	74.89 c	699.78 c	0.19 a		
N90	3786.35 b	79.51 a	40.02 a	58.65 a	72.65 a	141.20 a	37.51 a	190.68 c	59.98 a	81.89 b	762.08 b	0.20 a		
N150	4255.96 a	78.41 a	37.97 b	49.54 b	66.52 b	133.65 b	39.87 a	256.60 a	47.22 c	85.01 a	794.79 a	0.19 a		
N210	4268.67 a	72.45 b	32.79 c	46.70 c	58.97 c	143.01 a	38.34 a	232.96 b	46.21 c	81.30 b	752.73 b	0.18 a		
	**Non-Essential Amino Acids (μg/g** **)**													
**Nitrogen Application**	**Arg**	**Gln**	**Glu**	**Asn**	**Ala**	**Pro**	**Tyr**	**GABA**	**Gly**	**Asp**	**Ser**	**Orn**	**NEAA**	**EAA** **/NEAA**
N0	883.61 b	532.02 c	827.60 b	44.57 b	123.01 d	94.87 c	97.51 b	91.74 b	34.74 d	152.58 c	70.00 b	17.27 a	2969.53 b	0.24 ab
N90	813.77 c	560.90 c	838.86 b	57.85 a	138.90 c	107.39 b	105.20 a	85.67 c	42.12 c	175.91 b	82.22 a	15.48 a	3024.27 b	0.25 a
N150	974.19 a	650.69 b	1012.82 a	33.02 c	154.89 a	124.78 a	92.02 c	103.78 a	48.91 b	174.83 b	83.07 a	8.14 c	3461.16 a	0.23 ab
N210	990.87 a	707.87 a	1008.09 a	38.31 c	142.80 b	109.93 b	89.90 c	107.09 a	54.36 a	185.01 a	69.51 b	12.21 b	3515.94 a	0.21 b

Note: Amino acid concentrations are presented: Val (valine), Thr (threonine), Ile (isoleucine), Leu (leucine), Lys (lysine), Met (methionine), His (histidine), Phe (phenylalanine), Trp (tryptophan), EAA (essential amino acids), Arg (arginine), Gln (glutamine), Glu (glutamic acid), Asn (asparagine), Ala (alanine), Pro (proline), Tyr (tyrosine), GABA (gamma-aminobutyric acid), Gly (glycine), Asp (aspartic acid), Ser (serine), Orn (ornithine), NEAA (non-essential amino acids), EAA/NEAA (essential amino acids/non-essential amino acids). * means essential amino acids. Different small letters in the same column mean significant difference at *p* < 0.05 using the Tukey multiple-comparison test.

## Data Availability

The original contributions presented in the study are included in the article/Supplementary Materials, further inquiries can be directed to the corresponding author/s.

## Supplementary Materials

The following supporting information can be downloaded at: https://www.mdpi.com/article/10.3390/jof10070504/s1, Figure S1: Co-occurrence networks of bacterial (a,b) and fungal (c,d) communities in seed endophyte communities under different N application rates. a and c represent annotated nodes of bacteria and fungi by species, respectively, and b and d represent annotated nodes of bacteria and fungi by composition proportion of the group, respectively. The networks were constructed based on Spearman correlation analysis of taxonomic profiles; *p* < 0.05. Node size is proportional to relative abundance, and node colors indicate taxa at the order level. Internode connection (edge) indicates that there is a correlation between the two nodes being connected. Table S1: Precipitation and average temperature during quinoa growth stage. Table S2: Primers used in this study.

## References

[1] Risi J., Galwey N.W. (1984). The Chenopodium Grains of the Andes: Inca Crops for Modern Agriculture. Adv. Appl. Biol..

[2] Pasko P., Barton H., Zagrodzki P., Izewska A., Krosniak M., Gawlik M., Gawlik M., Gorinstein S. (2010). Effect of Diet Supplemented with Quinoa Seeds on Oxidative Status in Plasma and Selected Tissues of High Fructose-Fed Rats. Plant Foods Hum. Nutr..

[3] White P.L., Alvistur E., White H.S., Collazos C. (1955). Nutritive Values of Crops, Nutrient Content and Protein Quality of Quinua and Caihua, Edible Seed Products of the Andes Mountains. J. Agric. Food Chem..

[4] Jacobsen S.-E., Mujica Á., Jensen C.R. (2003). The Resistance of Quinoa (*Chenopodium quinoa* Willd.) to Adverse Abiotic Factors. Food Rev. Int..

[5] Zurita-Silva A., Fuentes F., Zamora P., Jacobsen S.-E., Schwember A.R. (2014). Breeding quinoa (*Chenopodium quinoa* Willd.): Potential and perspectives. Mol. Breed..

[6] Wright K., Pike O., Fairbanks D., Huber C. (2002). Composition of Atriplex hortensis, sweet and bitter Chenopodium quinoa seeds. J. Food Sci..

[7] Bastidas E., Roura R., Rizzolo D., Massanés T., Gomis R. (2016). Quinoa (*Chenopodium quinoa* Willd), from nutritional value to potential health benefits: An integrative review. J. Nutr. Food Sci..

[8] Liu X., Hu B., Chu C. (2022). Nitrogen assimilation in plants: Current status and future prospects. J. Genet. Genom..

[9] Lu Y., Gao L., Hu J., Liu X., Jiang D., Cao W., Dai T., Tian Z. (2024). Low nitrogen priming im proves nitrogen uptake and assimilation adaptation to nitrogen deficit stress in wheat seedling. Planta.

[10] Govindasamy P., Muthusamy S.K., Bagavathiannan M., Mowrer J., Vadivel R., Das T.K. (2023). Nitrogen use efficiency—A key to enhance crop productivity under a changing climate. Front. Plant Sci..

[11] Wang Z., Li N., Wang W., Zhu Y., Liu Y. (2023). Endophytic bacterial community diversity in genetically related hybrid rice seeds. Appl. Microbiol. Biotechnol..

[12] Kang X., Shen B., Wang H., Zhang J., Hu J., Guo M., Li Z., Chen X., Ma S., Yuan H. (2017). Effect of Different Amount Nitrogen Fertilizer and Ratio of Basic and Additional Fertilizers on Quinoa’s Yield and Economic Characters. J. Agric..

[13] Ni R., Zhang Y., Pang C., Wu R., Zhang Z., Tian Y., Liu L. (2015). Plastic Responses of Quinoa Seedling to Change of Water and Nitrogen Coupling. Crops.

[14] Petrini O. (1991). Fungal endophytes of tree leaves. Microbial Ecology of Leaves.

[15] Strobel G., Daisy B., Castillo U., Harper J. (2004). Natural products from endophytic microorganisms. J. Nat. Prod..

[16] Geisseler D., Scow K.M. (2014). Long-term effects of mineral fertilizers on soil microorganisms—A review. Soil Biol. Biochem..

[17] Dai Z., Su W., Chen H., Barberán A., Zhao H., Schadt C.W., Chang S.X. (2018). Long-term nitrogen fertilization decreases bacterial diversity and favors the growth of Actinobacteria and Proteobacteria in agro-ecosystems across the globe. Glob. Chang. Biol..

[18] Kavamura V.N., Hayat R., Clark I.M., Rossmann M., Mendes R., Hirsch P.R., Mauchline T.H. (2018). Inorganic nitrogen application affects both taxonomical and predicted functional structure of wheat rhizosphere bacterial communities. Front. Microbiol..

[19] Jin Y., Zhang K., Zhang X., Du J. (2009). Determination of amylose and amylopectin in wheat and wheat bud by dual wavelength method. J. Chin. Cereals Oils Assoc..

[20] (2016). National Food Safety Standard—Determination of Protein in Foods.

[21] (2016). National Standard for Food safety, the Determination of Fat in Food Products.

[22] (2016). Determination of Ash in National Standard for Food Safety.

[23] Bolyen E., Rideout J.R., Dillon M.R., Alexander H., Alm E.J., Arumugam M., Asnicar F. (2018). QIIME 2: Reproducible, interactive, scalable, and extensible microbiome data science. PeerJ Prepr..

[24] R Core Team, R (2011). A Language and Environment for Statistical Computing. Computing.

[25] Anderson M.J., Ellingsen K.E., McArdle B.H. (2006). Multivariate dispersion as a measure of beta diversity. Ecol. Lett..

[26] Segata N., Izard J., Waldron L., Gevers D., Miropolsky L., Garrett W.S., Huttenhower C. (2011). Metagenomic biomarker discovery and explanation. Genome Biol..

[27] Luo F., Zhong J., Yang Y., Scheuermann R.H., Zhou J. (2006). Application of random matrix theory to biological networks. Phys. Lett. A.

[28] Zhang L., Zhou X., Gu Q., Liang M., Mu S., Zhou B., Huang F., Lin B., Zou C. (2019). Analysis of the correlation between bacteria and fungi in sugarcane tops silage prior to and after aerobic exposure. Bioresour. Technol..

[29] Feng X., Pan L., Wang C., Zhang H. (2018). Residue analysis and risk assessment of pyrethrins in open fiel d and greenhouse turnips. Environ. Sci. Pollut. Res..

[30] Zhang Q., Acuña J.J., Inostroza N.G., Mora M.L., Radic S., Sadowsky M.J., Jorquera M.A. (2019). Endophytic Bacterial Communities Associated with Roots and Leaves of Plants Growing in Chilean Extreme Environments. Sci Rep..

[31] Zhang M., Zeiss M.R., Geng S. (2015). Agricultural pesticide use and food safety: California’s model. J. Integr. Agric..

[32] Cycoń M., Piotrowska-Seget Z. (2016). Pyrethroid-degrading microorganisms and their potential for the bioremediation of contaminated soils: A review. Front. Microbiol..

[33] Pino-Otín M.R., Val J., Ballestero D. (2019). Ecotoxicity of a new biopesticide produced by Lav andula luisieri on non-target soil organisms from different trophic levels. Sci. Total Environ..

[34] Ipsilantis I., Samourelis C., Karpouzas D.G. (2012). The impact of biological pesticides on arbuscular mycorrhizal fungi. Soil Biol. Biochem..

[35] Igarashi Y., Iida T., Yoshida R., Furumai T. (2002). Pteridic acids aand b, novel plant growth promoters with auxin-like activityfrom Streptomyces hygroscopicus TP-A0451. J. Antibiot..

[36] Pullen C.B., Schmitz P., Meurer K. (2002). New bioactive compounds from Streptomyces strains residing in the wood of Celastraceae. Planta.

[37] Mccormick M.H., Mcguire J.M., Pittenger G.E. (1955). Vancomycin, a new antibiotic. Chemical and biologic properties. Antibiot. Annu..

[38] Zhang Y., Liao Y., Chen S.W. (2010). Isolation, preliminaryidentification and nitrogen-fixation activity of endophytesfrom roots of one- and two-year-old Xanthoceras sorbifoliaplants. Chin. J. Plant Ecol..

[39] Gopal M., Gupta A., Arunachalam V. (2007). Impact of azadirachtin, an insecticidal allelochemi cal from neem on soil microflora, enzyme and respiratory activities. Bioresour. Technol..

[40] Kiran U., Patra D.D. (2003). Medicinal and aromatic plant materials as nitrification inhibitors for au gmenting yield and nitrogen uptake of Japanese mint (*Mentha arvensis* L. Var. Piperascens). Bioresour. Technol..

[41] Topp E., Mulbry W.M., Zhu H., Nour S.M., Cuppels D. (2000). Characterization of s-triazine herbicide metabolism by a Nocardioides sp. isolated from agricultural soil. Appl. Environ. Microbiol..

[42] Walvekar V.A., Bajaj S., Singh D.K. (2017). Ecotoxicological assessment of pesticides and their combination on rhizospheric microbial community structure and function of Vigna radiata. Environ. Sci. Pollut. Res..

