# Supplementary material for: Effects of Nitrogen Accumulation, Transportation, and Grain Nutritional Quality and Advances in Fungal Endophyte Research in Quinoa (Chenopodium quinoa Willd.) Plants
Source: J Fungi (Basel). 2024 Jul 21;10(7):504. doi: 10.3390/jof10070504 (PMC11277952; doi:10.3390/jof10070504)
Supplement: Supplementary file 1 [file jof-10-00504-s001.zip › jof-3093490-supplementary.pdf]

Supplementary Table S1. Precipitation and average temperature during quinoa growth stage

Growth stage	Sowing - Seedling stage	Seedling - Branchin g stage	Branchin g – Sprouting stage	Sprouting - flowering stage	Flowerin g - Filling stage	Filling - Mature stage	Growing days
Date	4.10-5.10	5.10-6.1	6.1-6.18	6.18-6.27	6.27-8.2	8.2-8.22	135 d
Precipitation （mm）	91.86	81.69	65.98	25.07	110.67	95.04	Total precipitation 378.45
Average temperature （℃）	12.21	10.56	16.60	22.97	25.57	22.85	——

Supplementary Table S2. Primers used in this study

Primer	DNA sequence (5′→3′)
Forward primer for V5-V7 region of 16S rRNA	AACMGGATTAGATACCCKG
Reverse primer for V5-V7 region of 16S rRNA	ACGTCATCCCCACCTTCC
Forward primer for fungal ITS1-2 region	CTTGGTCATTTAGAGGAAGTAA
Reverse primer for fungal ITS1-2 region	GCTGCGTTCTTCATCGATGC

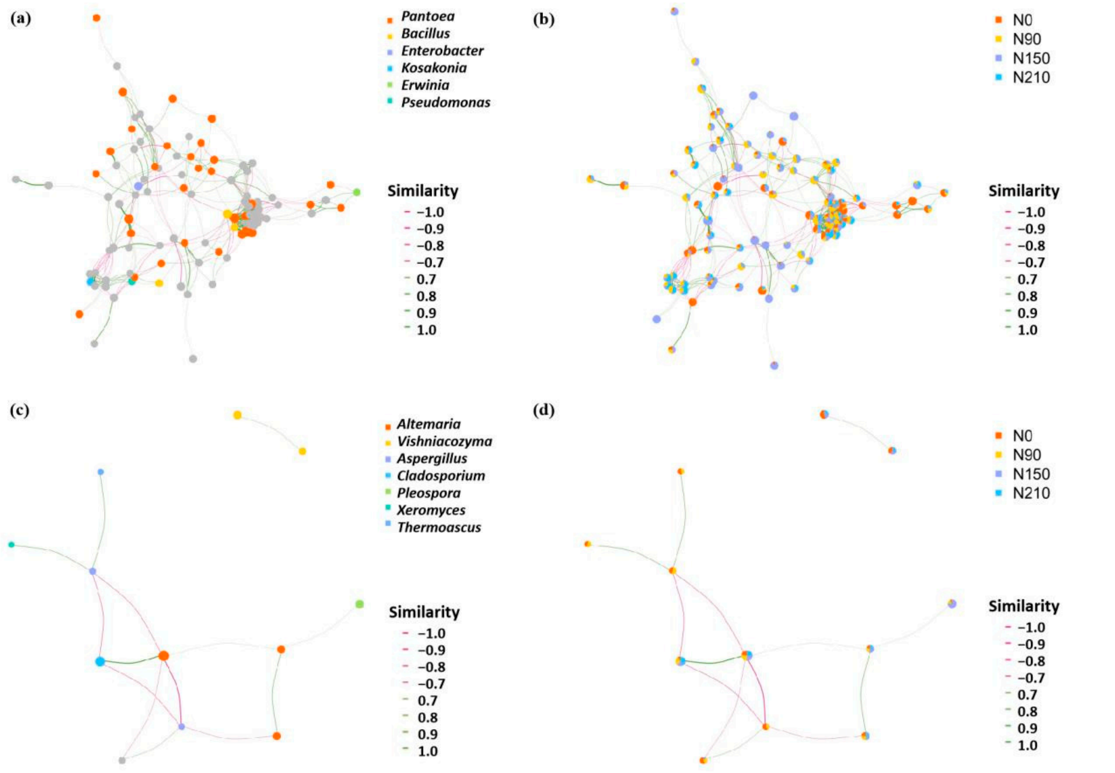

Figure S1. Co-occurrence networks of bacterial (a,b) and fungal (c,d) communities in seed endophyte communities under different N application rates. a and c represent annotated nodes of bacteria and fungi by species, respectively, and b and d represent annotated nodes of bacteria and fungi by composition proportion of the group, respectively. The networks were constructed based

on Spearman correlation analysis of taxonomic profiles; $p < 0.05$. Node size is proportional to relative abundance and node colors indicate taxa at the order level. Internode connection (edge) indicates that there is a correlation between the two nodes being connected.
